# The *Mitragyna speciosa* (Kratom) Genome: a resource for data-mining potent pharmaceuticals that impact human health

**DOI:** 10.1093/g3journal/jkab058

**Published:** 2021-03-02

**Authors:** Julia Brose, Kin H Lau, Thu Thuy Thi Dang, John P Hamilton, Lívia do Vale Martins, Britta Hamberger, Bjoern Hamberger, Jiming Jiang, Sarah E O’Connor, C Robin Buell

**Affiliations:** 1 Department of Plant Biology, Michigan State University, East Lansing, MI 48824, USA; 2 Department of Chemistry, University of British Columbia Okanagan, Kelowna, BC V1V 1V7, Canada; 3 Departamento de Genética, Universidade Federal de Pernambuco, Recife, PE 50670-901, Brazil; 4 Department of Biochemistry & Molecular Biology, Michigan State University, East Lansing, MI 48824, USA; 5 Department of Horticulture, Michigan State University, East Lansing, MI 48824, USA; 6 MSU AgBioResearch, Michigan State University, East Lansing, MI 48824, USA; 7 Department of Natural Product Biosynthesis, Max Planck Institute for Chemical Ecology, D-07745 Jena, Germany; 8 Plant Resilience Institute, Michigan State University, East Lansing, MI 48824, USA

**Keywords:** kratom, Mitragyna speciosa, genome, alkaloids, linked-read assembly

## Abstract

*Mitragyna speciosa* (kratom) produces numerous compounds with pharmaceutical properties including the production of bioactive monoterpene indole and oxindole alkaloids. Using a linked-read approach, a 1,122,519,462 bp draft assembly of *M. speciosa* “Rifat” was generated with an N50 scaffold size of 1,020,971 bp and an N50 contig size of 70,448 bp that encodes 55,746 genes. Chromosome counting revealed that “Rifat” is a tetraploid with a base chromosome number of 11, which was further corroborated by orthology and syntenic analysis of the genome. Analysis of genes and clusters involved in specialized metabolism revealed genes putatively involved in alkaloid biosynthesis. Access to the genome of *M. speciosa* will facilitate an improved understanding of alkaloid biosynthesis and accelerate the production of bioactive alkaloids in heterologous hosts.

## Introduction

**Figure 1:**
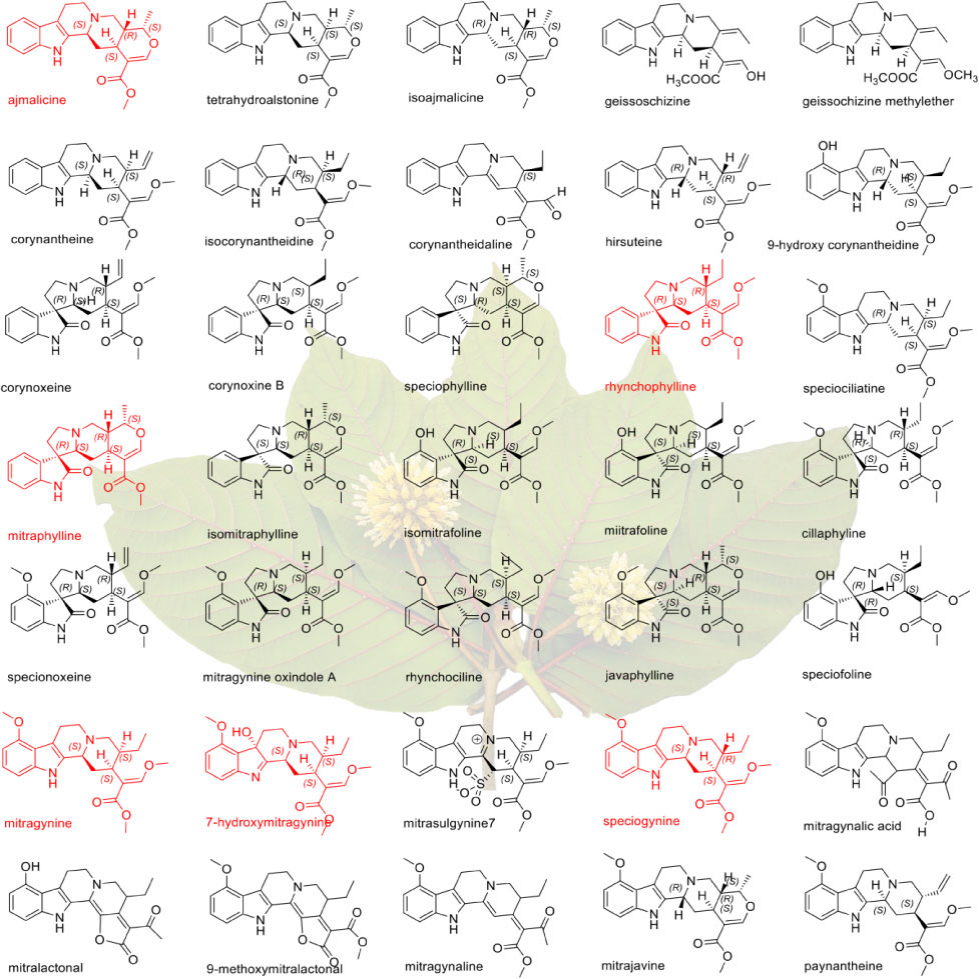
Structural diversity of alkaloids from *M. speciosa*. Red structures signify compounds with reported pharmaceutical properties. Photo credit: Jade at the Healing East.

The Rubiaceae is one of the largest families of angiosperms with an estimated 13,000 species within 650 genera (https://www.mobot.org/mobot/research/apweb). The family is well-known for its specialized metabolism, of which, a number of species have been cultivated for human use. The most well-known and economically important genus is *Coffea* (coffee) known for its stimulatory alkaloid caffeine (Figure 1). Consequently, the Rubiaceae is often referred to as the “coffee family”. Several other species in this family are of commercial or pharmaceutical relevance, including many important alkaloid-producing species such as *Theobroma cacao* (heart stimulant theobromine), *Cinchona officinalis* (antimalarial quinine), and *Carapichea ipecacuanha* (expectorant ipecac), formerly known as *Psychotria ipecacuanha* (Achan *et al.* 2011). The Rubiaceae also contains ornamentals including *Gardenia*, which are prized for their fragrance attributable to the production of volatile specialized metabolites (Liu and Gao 2000) and *Rubia tinctorum* (madder), which has been used for its red coloring properties. Moreover, for a variety of Rubiaceae species, aphrodisiac or psychoactive properties have been reported (Adkins *et al.* 2011). The species *Uncaria tomentosa* (Cat’s claw) and *Mitragyna speciosa* (kratom; Figure 1), have been used for centuries in China, Southeast Asia, and South America as folk medicines (Erowele and Kalejaiye 2009; Adkins *et al.* 2011). In a majority of the studies, the biological activity of these species is attributed to unique monoterpene indole and oxindole alkaloids.

*M. speciosa* is native to Southeast Asia and traditionally was used to combat fatigue, treat pain, as a relaxant, and as a stimulant (Suwanlert 1975). However, *M. speciosa* has emerged in recent years as an herbal remedy to treat not only pain, but also to alleviate symptoms associated with opiate withdrawal as well as use as a psychostimulant (Boyer *et al.* 2008; McWhirter and Morris 2010; Nelsen *et al.* 2010). *M. speciosa* exhibits dose-dependent responses with low doses providing stimulatory effects similar to cocaine and amphetamines, whereas high doses lead to sedative and narcotic effects (Prozialeck *et al.* 2012). While *M. speciosa* is reported to produce numerous different alkaloids (Brown and Alper 2017), the pharmacological effects are attributed to monoterpene indole alkaloids (MIAs), specifically, mitragynine, and 7-hydroxymitragynine. Not surprisingly, due to its structural similarity to opioids, mitragynine has been shown to act on the μ, κ, and δ opioid receptors and is a potent opioid agonist (Thongpradichote *et al.* 1998; Takayama 2004) whereas the oxindole mitraphylline has been shown to have promising anti-cancer activity (Bacher *et al.* 2006; Bigliani *et al.* 2013; Kaiser *et al.* 2013). Other compounds have been isolated from *M. speciosa* and reported to have pharmaceutical applications including rhynchophylline (noncompetitive *N*-methyl-d-aspartate receptor antagonist), speciophylline, speciogynine (smooth muscle relaxer), and paynatheine (stimulant) (Figure 1; Shellard *et al.* 1978). These compounds have been shown to modulate intestinal smooth muscle function and behavioral response in animals (Matsumoto *et al.* 2005).

Currently, *M. speciosa* is banned in a number of countries but available as an herbal remedy in certain countries such as the United States and Canada. While the safety of *M. speciosa* has been questioned, the US Drug Enforcement Agency withdrew a notice of intent to classify mitragynine and 7-hydroxymitragynine as Schedule I drugs pending the outcome of an investigation by the US Food and Drug Administration which issued a public health advisory on the use of *M. speciosa* (O’Malley 2018). Understanding the biosynthesis of the bioactive alkaloids in *M. speciosa* would permit heterologous expression of individual compounds thereby enabling more precise pharmacological studies on the positive and/or negative outcomes of this medicinal plant and eventually, informed breeding and production strategies for this species. In this study, we report on the draft genome sequence and annotation of *M. speciosa*, demonstrate that *M. speciosa* is a tetraploid, and has conserved loci involved in specialized metabolism that can be harnessed to identify loci involved in MIA biosynthesis.

## Materials and methods

### Plant material and chromosome analysis

*M. speciosa* “Rifat” was purchased as rooted cuttings from World Seeds Supply (https://www.worldseedsupply.com/) and grown in a greenhouse at Michigan State University (East Lansing, MI, USA) at 22°C day/18°C night. From November to April, the greenhouse was supplemented with 12 hours of light. For mitotic metaphase chromosome preparation, root tips were harvested from a greenhouse-grown “Rifat” plant and treated with nitrous oxide at a pressure of 160 psi (∼10.9 atm) for 40 minutes (Braz *et al.* 2018). Root tips were then fixed in three methanol: one glacial acetic acid for 24 hours at room temperature and stored at −20°C until use. Meristems were digested with an enzymatic solution containing 2% pectolyase (Sigma-Aldrich, St Louis, MO, USA), 4% cellulase (Yakult Pharmaceutical, Tokyo), and 20% pectinase (Sigma-Aldrich, St Louis, MO, USA) for 2 hours at 37°C and slides prepared as described previously with minor modifications (De Carvalho and Saraiva 1993). Chromosomes were counterstained with DAPI (4',6-diamidino-2-phenylindole) in VECTASHIELD antifade solution (Vector Laboratories, Burlingame, CA, USA). Metaphase images were captured using a QImaging Retiga EXi Fast 1394 CCD camera attached to an Olympus BX51 epifluorescence microscope and processed with Meta Imaging Series 7.5 software. The final image was optimized for brightness and contrast with Adobe Photoshop CS4 (Adobe Systems Incorporated) software.

### Nucleic acid isolation, library construction, and sequencing

Genomic DNA was isolated from young “Rifat” leaves from a single plant using a modified cetrimonium bromide protocol that includes a sorbitol buffer wash step to remove polysaccharides (Tel-zur *et al.* 1999). A single 10x Genomics long read library (Chromium Genome Reagent v2 kit; 10x Genomics, Pleasanton, CA) was constructed at the Van Andel Institute and sequenced on the HiSeq 4000 in the Research Technology Support Facility (RTSF) Genomics Core at Michigan State University. A separate Illumina compatible whole genome shotgun (WGS) sequencing library was constructed as described previously (Hardigan *et al.* 2016) and sequenced in paired-end mode on a HiSeq4000 at the RTSF Genomics Core at Michigan State University generating 150 nt reads.

Immature leaves, mature leaves, leaf bracts, roots, stems, petioles, and leaves 6 days after wounding from greenhouse-grown plants were harvested and flash frozen in liquid nitrogen (Supplementary Table S1). RNA was isolated using the method described previously (Kolosova *et al.* 2004) with these modifications: the amount of tissue was scaled down to 100 mg and the RNA pellet was washed with 70% ethanol following LiCl precipitation prior to resuspension in nuclease-free water. After DNAse treatment (DNA-freeT Kit; Ambion, Austin, TX, USA), RNA integrity was assessed using the RNA 6000 Nano kit (Bioanalyzer 2100; Agilent, Santa Clara, CA, USA). For gene annotation, mature leaf and root RNA were used to construct KAPA Stranded RNA-Sequencing (RNA-Seq) libraries using NEB adapters and primers (Roche Sequencing, San Diego, CA, USA) and sequenced in paired-end mode on the HiSeq2500 at the RTSF Genomics Core at Michigan State University. For expression abundance estimations, RNA-seq libraries were constructed using the Illumina TruSeq kit (Stranded mRNA—polyA mRNA; Illumina) and single-end 50 nt reads were generated at the RTSF Genomics Core at Michigan State University on the HiSeq4000. All sequencing materials and sequencing strategies are listed in Supplementary Table S1.

### Genome assembly and scaffold filtering

The “Rifat” genome was assembled with Supernova v2.0.1 (Weisenfeld *et al.* 2017) using 631 M 151 nt reads from a single 10x library, equivalent to 77.59x raw coverage and 52.89x effective coverage after accounting for duplicated reads, as calculated by Supernova. The genome assembly was extracted from the raw assembly output using the Supernova mkoutput function creating two assembly files: one with the pseudohap1 style and the other with the pseudohap2 style, both with a minimum scaffold size of 500 nt. Downstream analyses were conducted on the pseudohap1 assembly. Redundant scaffolds were removed using the redundancy reduction module of Redundans (v0.14a; Pryszcz and Gabaldón 2016) with an identity of 99, overlap of 95, minimum length of 5 kb, no scaffolding, and no gap-closing options. Mean scaffold read depth values were calculated from alignments of the 10x library using BWA-MEM (bwa v0.7.12; Li 2013) with the –M option followed by removal of duplicate reads using MarkDuplicates (picardTools v2.7.2; http://broadinstitute.github.io/picard/) and calculated by dividing the total read bases aligned to each scaffold, as reported by SAMtools (v1.4; Li *et al.* 2009) bedcov, by the length of each scaffold minus gaps. The distribution of per-base depth of coverage was calculated using SAMtools depth with the parameters –aa and –d 0.

Scaffolds in the filtered 10x assembly were queried against the National Center for Biotechnology Information nucleotide database (NCBI; downloaded May 1, 2018) using BLASTN (BLAST+ v2.6.0; Camacho *et al.* 2009) with the parameter –max_target_seqs 100000. Using filters of *E*-value <e-40, Query Coverage Per Subject >90, Query Coverage Per HSP >50 and identity >90; one nonViridiplantae scaffold (6 kb) was detected. Further investigation revealed that this scaffold was the PhiX sequencing control (NC_001422.1). To remove chloroplast genome scaffolds, the scaffolds were queried against Rubiaceae chloroplast genomes downloaded from NCBI (Supplementary Table S2). BLASTN filters for chloroplast scaffolds were Query Coverage Per Subject >97, Query Coverage Per HSP >50 and identity >97; six chloroplast scaffolds, totaling 153 kb, were removed.

### Genome assembly quality assessment

Standard sequence content and contiguity metrics were obtained using assemblathon_stats.pl from Assemblathon (v2; Bradnam *et al.* 2013). BUSCO 3 (v3.1.0; Simao *et al.* 2015) was run using the embryophyta_odb9 database (1440 BUSCO groups) in genome mode. Genomic DNA reads were cleaned of adaptors and low-quality bases using Cutadapt (v1.18; Martin 2011) and aligned to the genome using BWA-MEM (bwa v0.7.12; Li 2013) with the –M option followed by removal of duplicate reads using MarkDuplicates (picardTools v2.7.2; http://broadinstitute.github.io/picard/). RNA-Seq reads were cleaned of adaptors and low-quality bases using Cutadapt (v1.18; Martin 2011) and aligned to the genome using HISAT2 (v2.1.0; Kim *et al.* 2015) with options q, max-intronlen 5000, and new-summary. Alignment metrics were then obtained using SAMtools flagstat and Picard CollectAlignmentSummaryMetrics (picardTools v2.9.2; http://broadinstitute.github.io/picard/).

### Gene annotation

A *M. speciosa* “Rifat” custom repeat library was created using RepeatModeler (v1.0.8; http://repeatmasker.org) and matched against a curated library of plant protein-coding genes and sequences identified using ProtExcluder (v1.1; Campbell *et al.* 2014). The resulting repetitive sequences were combined with RepBase Viridiplantae repeats (v20150807; Jurka *et al.* 2005) to create a final custom repeat library. The assembly was then masked using RepeatMasker (v4.0.6; http://repeatmasker.org; Chen 2004) using the custom repeat library with the –s, –nolow, and –no_is options; subsequent gene annotations were derived from the masked genome.

The paired-end RNA-Seq libraries were aligned using TopHat2 (v2.1.1; Kim *et al.* 2013) with the parameters–min-intron-length 20 and –max-intron-length 20000 in stranded mode. *Ab initio* gene prediction was performed by training AUGUSTUS (v3.2.2; Stanke *et al.* 2006), using the hints provided by the alignments of the leaf RNA-seq library and the soft-masked genome assembly. Genome-guided transcript assemblies were constructed using Trinity (v2.3.2; Grabherr *et al.* 2011) with the parameters –genome_guided_max_intron 20000 and –SS_lib_type RF, removing transcripts shorter than 500 bp. Gene predictions were then refined using PASA2 (v2.0.2; https://github.com/PASApipeline/PASApipeline/wiki; Haas *et al.* 2008), utilizing genome-guided transcript assemblies as evidence to yield the working gene model set.

High-confidence gene models were defined within the working gene model set by several criteria. First, transcripts must be flanked by start and stop codons, with no internal stop codon. Second, it must have a PFAM (v31; Finn *et al.* 2016) hit with an *E*-value ≤1e-5 and a domain *E*-value ≤1e-3 as identified by HMMER (v3.1b2; Mistry *et al.* 2013) or have a FPKM value greater than 0 in any of the single-end RNA-seq libraries, as calculated using Cufflinks (v2.2.1; Trapnell *et al.* 2010) with the parameters –multi-read-correct, –frag-bias-correct, –max-bundle-frags 999999999, and –max-intron-length 20000 in stranded mode. Third, it must not have the best PFAM hit to a transposable element-related domain. Functional annotations were inferred from BLASTP (BLAST+ v2.6; Camacho *et al.* 2009) queries against *Arabidopsis thaliana* (Araport 11; Cheng *et al.* 2017) and manually curated Viridiplantae entries in Swiss-Prot (downloaded December 12, 2019; Boeckmann *et al.* 2003), filtering for an E-value ≤1e-5. Gene ontology terms were annotated using InterProScan (v5.28.67.0; Jones *et al.* 2014).

### Comparative analyses

The longest peptide for each gene was obtained for *Amborella trichopoda* (v1), *A. thaliana* (Araport 11), *Coffea canephora, Solanum lycopersicum* (ITAG2.4), *T. cacao* (v1.1), *Vitis vinifera* (Genoscope.12X), and the high-confidence gene set for *M. speciosa*. All datasets were downloaded from Phytozome (v12.1; Goodstein *et al.* 2012) except *C. canephora* (http://coffee-genome.org/) and *M. speciosa*. Orthologous groups were identified using Orthofinder (v2.2.7; Emms and Kelly 2019) with the parameters –S blast and –M msa. BLASTP (BLAST+ v2.6.0; Camacho *et al.* 2009) was run for the longest peptide per gene for *M. speciosa* and *C. canephora*, making self-comparisons and cross-comparisons in both directions. The top five hits for each query were retained after filtering with an *E*-value <1e-5. Visualization of the orthogroup membership was performed using the package UpsetR (Conway *et al.* 2017) in R (https://www.r-project.org/).

### Monoterpene indole alkaloid biosynthetic pathway

Mitragynine and mitraphylline are derived from the central MIA intermediate strictosidine and using validated genes from the MIA-producing species *Catharanthus roseus* (Kellner *et al.* 2015), putative orthologs of the methylerythritol phosphate and iridoid pathways were identified as well as the downstream genes strictosidine synthase and tryptophan decarboxylase in *M. speciosa.* A BLASTP search (BLAST+ v2.6; Camacho *et al.* 2009) of the working set of *M. speciosa* genes with the options E value ≤1e-40, query coverage ≥70, and percent identity ≥50 was used to identify putative orthologs. The expression of the putative orthologs was determined using Cufflinks (v2.2.1; Trapnell *et al.* 2012) on the working set of genes with the –b option.

## Results and discussion

### Genome assembly of *M. speciosa*

**Figure 2:**
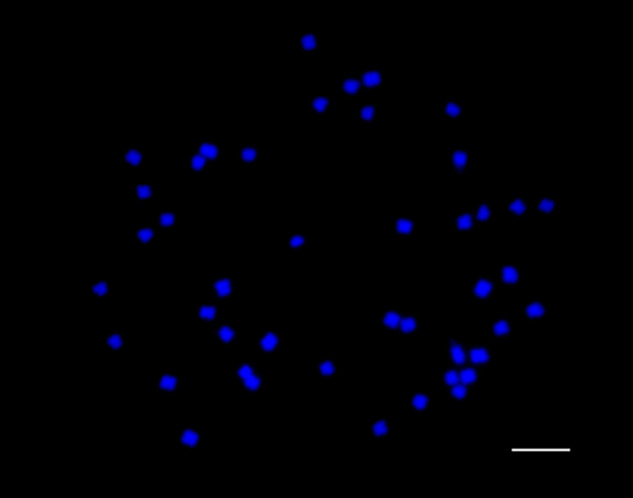
Mitotic metaphase chromosomes of *M. speciosa*. Digested meristems from root tips counterstained with DAPI pictured in blue. Bar = 5 μm.

A linked-read approach was employed to generate a genome assembly of *M. speciosa* “Rifat.” Using 631,344,782 reads representing 88 Gb of sequence generated from a single 10x Chromium library, the *M. speciosa* “Rifat” genome was assembled using Supernova (Weisenfeld *et al.* 2017). The raw Supernova assembly was composed of 36,453 scaffolds totaling 1,187,578,907 bp with an N50 scaffold length of 879,459 bp (Table 1). Scaffolds were filtered to remove small scaffolds (<5 kb) plastid sequences, contaminants, and haplotig sequences. The final assembly was 1,122,519,462 bp (968,285,288 bp ungapped sequence) located on 17,031 scaffolds with an N50 scaffold size of 1,020,971 bp, a maximum scaffold size of 9,844,214 bp, and an N50 contig size of 70,448 bp (Table 1). The genus *Mitragyna* is reported to be a tetraploid with a base chromosome number of 11 (Kiehn 1995). Chromosome counts of *M. speciosa* “Rifat” root tips revealed 2*n *=* *4*x *=* *44 chromosomes (Figure 2), consistent with “Rifat” being a tetraploid. Flow cytometry of “Rifat” leaves revealed a 2 C size of 1.56 Gb (1 C = 780 Mb) whereas the ungapped assembly size is 968 Mb suggesting that residual haplotigs remain in the final assembly.

**Table 1:** Assembly metrics of *M. speciosa* “Rifat” assembly

Scaffolds	Initial assembly	Final assembly
Number of scaffolds	36,453	17,031
Total size of scaffolds (bp)	1,187,578,907	1,122,519,462
Longest scaffold (bp)	9,844,214	9,844,214
Shortest scaffold (bp)	1,000	5,001
Mean scaffold size (bp)	32,578	65,910
Median scaffold size (bp)	5,162	10,864
N50 scaffold length (bp)	879,459	1,020,971
L50 scaffold count	260	225
Scaffold %N	13.00%	13.74%
Ungapped size (bp)	1,033,245,372	968,312,152
**Contigs**		
Percentage of assembly in scaffolded contigs	76.30%	80.40%
Number of contigs	49,303	29,145
Number of contigs in scaffolds	16,150	14,757
Total size of contigs (bp)	1,033,348,707	968,424,462
Mean contig size (bp)	20,959	33,228
Median contig size (bp)	6,703	15,187
N50 contig length (bp)	63,984	70,448
Contig %N	0.01%	0.01%

Initial Assembly generated by Supernova; Final Assembly generated after filtering.

Quality assessments of the final assembly were performed to determine its representation of the genome and gene space. Alignment of three independent WGS sequencing datasets to the final assembly resulted in 95.9%–97.8% aligned reads, of which, 99.2%–99.7% were properly paired (Supplementary Table S3) suggesting accurate assembly of the genome. Read depth across the scaffolds were examined revealing that the majority of the scaffolds had a read depth of 55.4 (log_2_ 5.7) (Supplementary Figure S1); however, scaffolds with lower and higher read depth are present in the final assembly suggesting the presence of unpurged haplotigs as well as collapsed homeologs, respectively. To reveal the extent of unpurged haplotigs and collapsed scaffolds, the average depth of each scaffold was plotted (Supplementary Figure S1). This revealed two peaks; the first belonging to uncollapsed scaffolds and the second belong to collapsed scaffolds.

To assess the representation of genic space, leaf and root paired-end RNA-seq libraries were aligned to the assembly revealing an alignment of 95.1 and 93.6% of the reads (Supplementary Table S4), respectively, of which, 97.2 and 96.8% were properly paired, respectively. We also aligned additional single end RNA-Seq libraries from diverse tissues (13 samples, Supplementary Table S1) and observed alignment rates of 93.4–95.2% (Supplementary Table S4). We also assessed representation of conserved orthologs using the Benchmarking Universal Single-Copy Orthologs tool (Simao *et al.* 2015) revealing 88.5% complete orthologs with 4% fragmented (C: 88.5% [S: 45.4%, D: 43.1%], F: 4%, M: 7.5%, n: 1440). Not surprisingly, 43.1% of the BUSCO orthologs were present as duplicates, consistent with the reported tetraploid nature of *Mitragyna* species (Kiehn 1995) and a chromosome count of 44. Approximately 45.4% of the BUSCO orthologs were present in single-copy that could be due to loss of an ortholog in one of the two kratom subgenomes or due to the collapse of the two homeologs into a single scaffold in regions of the two subgenomes with high-sequence identity. Collectively, these data support a high-quality draft assembly of *M. speciosa*.

### Genome annotation

To annotate the genome, a custom repeat library was constructed and used to mask the assembly for repetitive sequences; in total, 44.18% of the genome was identified as repetitive sequences (Supplementary Table S5). The GC content was 34.49 and a total of 495,976,085 bases were masked. Paired end RNA-seq reads from leaf and root tissue were used to generate genome-guided transcript assemblies to train Augustus as described previously (Zhao *et al.* 2019). The initial Augustus-generated gene models were then refined using PASA2 (Haas *et al.* 2008) resulting in a working set of 70,611 genes encoding 93,399 gene models (Table 2). A high confidence gene model set was generated by first removing genes models that lack a start and stop codon or contain a Pfam domain related to transposable elements, and then identifying gene models that were either expressed (FPKM >0; Fragments per kb exon model per million mapped reads) or had a significant Pfam domain match; 55,746 genes encoding 77,857 gene models are in the high confidence gene set (Table 2).

**Table 2:** Genome annotation metrics for *M. speciosa* “Rifat”

	Working Set	High confidence Set
Number of genes	70,611	55,746
Number of transcripts	93,399	77,857
Mean transcript length (bp)	1,456.90	1,630.49
Mean gene length (bp)	2,992.50	3,439.99
Mean exon number	6.46	7.49
Mean CDS length (bp)	1,094.33	1,206.73

### Comparative analyses of *M. speciosa* with related species

**Figure 3:**
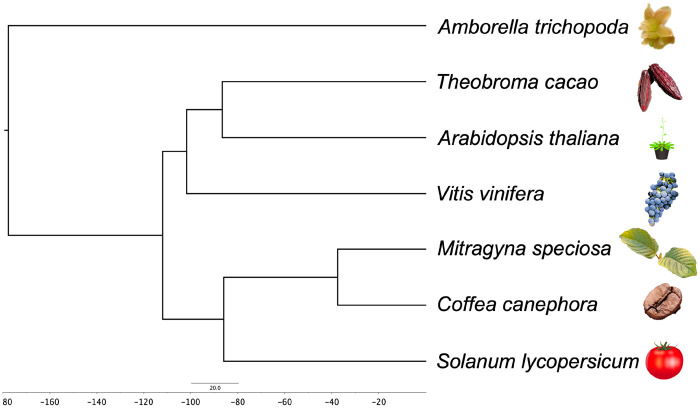
Phylogeny of *M. speciosa* and other angiosperms. Phylogeny was obtained from Orthofinder (v2.2.7; Emms and Kelly 2019). Photo credit: Sangtea Kim (*Amborella trichopoda* picture).

**Figure 4:**
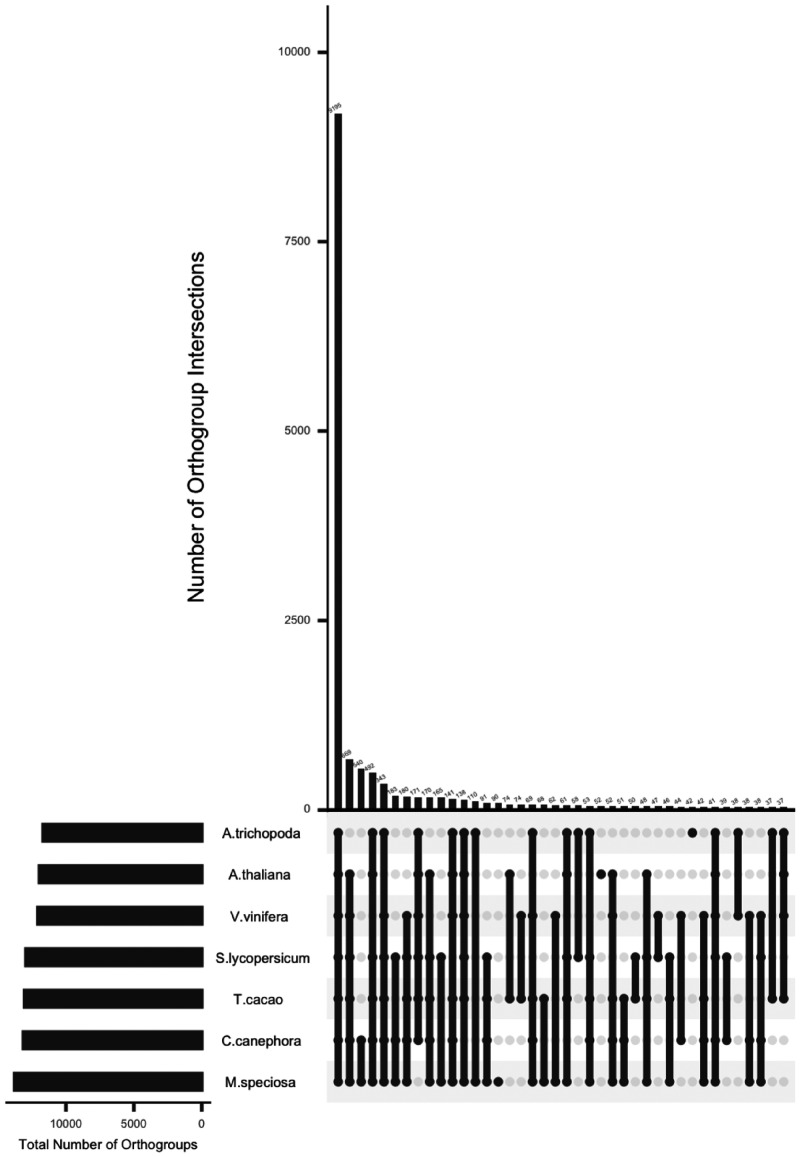
Orthogroups of *M. speciosa* from Orthofinder (v2.2.7; Emms and Kelly 2019). The number of orthogroups identified between *Amborella trichopoda, Arabidopsis thaliana, Vitis vinifera, Solanum lycopersicum, Theobroma cacao, Coffea canephora*, and *M. speciosa*. The numbers on top of each bar are the number of orthogroups that are present amount the species with black-filled circles below the *x*-axis. The proportion of the species present in orthogroups is shown to the left of the axis.

Using the predicted proteomes of *A. trichopoda* (DePamphilis *et al.* 2013), *A. thaliana* (Cheng *et al.* 2017), *C. canephora* (Denoeud *et al.* 2014), *T. cacao* (Argout *et al.* 2011), *S. lycopersicum* (Sato *et al.* 2012), *V. vinifera* (Jaillon *et al.* 2007), and *M. speciosa*, orthologous and paralogous groups were generated using OrthoFinder (v2.2.7; Emms and Kelly 2019); these relationships are presented in a phylogeny that is consistent with known relationships among these species (Figure 3). Clustering of these seven proteomes revealed 15,194 orthologous groups containing 55,542 genes; *M. speciosa* had 90 lineage-specific paralogous groups containing 479 genes (Figure 4).

**Figure 5:**
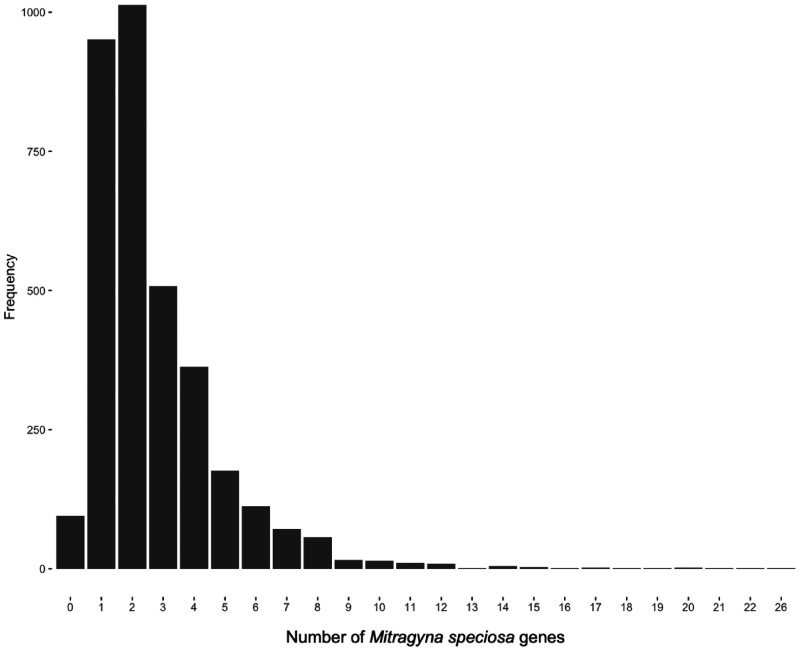
Frequency of orthogroups with various numbers of *M. speciosa* genes per one gene of other species. *Amborella trichopoda, Arabidopsis thaliana, Vitis vinifera, Solanum lycopersicum, Theobroma cacao*, and *Coffea canephora* are the species were only one gene is present in the orthogroups. Frequency refers to the number of orthogroups identified by Orthofinder (v2.2.7; Emms and Kelly 2019). Orthogroups are separated by the number of *M. speciosa* genes present when the orthogroup contains one gene from *A. trichopoda, A. thaliana, V. vinifera, S. lycopersicum, T. cacao*, and *C. canephora*.

Orthologous groups (3,415 total) containing a single *A. thaliana, A. trichopoda, S. lycopersicum, T. cacao, V. vinifera*, and *C. canephora* gene and therefore, putatively single copy genes across these species were examined for the number of *M. speciosa* genes within the orthogroup (Figure 5). Of the 3,415 orthogroups, 3% contained no *M. speciosa* genes, 28% of orthogroups had a one-to-one ratio throughout all species including *M. speciosa*, 30% of orthogroups contained two genes in *M. speciosa* per one gene of another species, and 39% contain three or more genes in *M. speciosa* per one gene of another species (Figure 5). The observation of increased duplicated genes in *M. speciosa* relative to the other species supports the tetraploid nature of *M. speciosa.* Orthogroups specific to the Rubiaceae species (*C. canephora* and *M. speciosa*) were also consistent with the tetraploid nature of *M. speciosa* as only 4% of the Rubiaceae-lineage specific orthogroups contained a single *C. canephora* gene not present in *M. speciosa*, 23% were present in a one-to-one ratio, 27% contained two duplicated genes in *M. speciosa* per one *C. canephora* gene, and 46% of orthogroups contain three or more genes in *M. speciosa* per one *C. canephora* gene (Supplementary Figure S2).

### Genes encoding specialized metabolism

A feature of some specialized metabolic biosynthetic pathways is gene co-expression and physical clustering in the genome (Nützmann *et al.* 2016). The *M. speciosa* genome was examined for candidate genes involved in biosynthesis of strictosidine, the central intermediate in MIA biosynthesis. Putative orthologs of *C. roseus* MIA pathway genes were identified for eight genes of the methylerythritol phosphate pathway, nine genes of the iridoid pathway, tryptophan decarboxylase, and strictosidine synthase within the working set of genes (Supplementary Table S6). As gene expression data is available for leaves (young and mature), roots, stems, petioles, bracts, and wounded leaves, coexpression analyses can be performed to decipher which of these putative orthologs function in MIA biosynthesis in kratom.

Some specialized metabolism pathways are physically clustered in plant genomes and Planti-SMASH (Kautsar *et al.* 2017) was used to identify clusters of specialized metabolism genes. In total, 72 clusters were identified (Supplementary Table S7). One cluster is predicted to encode alkaloid biosynthetic genes including a copper amine oxidase, epimerase, and cytochrome P450. The other predicted cluster types are terpene, saccharide-terpene, saccharide-alkaloid, saccharide, polyketide-alkaloid, polyketide, lignan-polyketide, lignan, alkaloid, and putative clusters.

## Conclusions

Access to an annotated genome assembly of *M. speciosa* “Rifat,” coupled with access to gene expression profiles, will facilitate the discovery of alkaloid biosynthetic pathway genes and heterologous production of bioactive alkaloids. Furthermore, the *M. speciosa* genome will aid in improving our understanding of the evolution of plant specialized metabolic pathways and provide a resource to understand genetic diversity in *M. speciosa*.
